# Characterization of the Immune Response to *Vibrio cholerae* Infection in a Natural Host Model

**DOI:** 10.3389/fcimb.2021.722520

**Published:** 2021-11-23

**Authors:** Dustin A. Farr, Dhrubajyoti Nag, Jeffrey H. Withey

**Affiliations:** Department of Biochemistry, Microbiology, and Immunology, Wayne State University School of Medicine, Detroit, MI, United States

**Keywords:** zebrafish, cholera, immune response, *Vibrio cholerae*, bacterial pathogenesis

## Abstract

The gram-negative bacterium *Vibrio cholerae* causes the life-threatening diarrheal disease cholera, which is spread through the ingestion of contaminated food or water. Cholera epidemics occur largely in developing countries that lack proper infrastructure to treat sewage and provide clean water. Numerous vertebrate fish species have been found to be natural *V. cholerae* hosts. Based on these findings, zebrafish (*Danio rerio*) have been developed as a natural host model for *V. cholerae.* Diarrheal symptoms similar to those seen in humans are seen in zebrafish as early as 6 hours after exposure. Our understanding of basic zebrafish immunology is currently rudimentary, and no research has been done to date exploring the immune response of zebrafish to *V. cholerae* infection. In the present study, zebrafish were infected with either pandemic El Tor or non-pandemic, environmental *V. cholerae* strains and select immunological markers were assessed to determine cellular immunity and humoral immunity. Significant increases in the gene expression of two transcription factors, T-bet and GATA3, were observed in response to infection with both *V. cholerae* strains, as were levels of mucosal related antibodies. Additionally, the cytokine IL-13 was shown to be significantly elevated and paralleled the mucin output in zebrafish excretions, strengthening our knowledge of IL-13 induced mucin production in cholera. The data presented here further solidify the relevancy of the zebrafish model in studying *V. cholerae*, as well as expanding its utility in the field of cholera immunology.

## Introduction

*Vibrio cholerae* is a gram-negative bacterium that causes the diarrheal disease cholera. Endemic to warmer climates such as Southeast Asia, Latin America, and parts of Africa, *V. cholerae* can be found in aquatic environments including fresh, salt or brackish water (Baker-Austin et al., 2013; Ali et al., 2015). Roughly 3-5 million people per year become infected with cholera, leading to estimates of ~140,000 deaths, with half of those being children aged 5 years or younger (Ali et al., 2015). With current advancements of climate change and warming temperatures, the spread of *V. cholerae* to new geographical areas leads to the urgent need for research furthering our understanding of this pathogen (Vezzulli et al., 2016; Deeb et al., 2018).

Strains of *Vibrio cholerae* that can cause pandemics and the disease cholera are those categorized in the O1 or O139 serogroups, while the majority of *V. cholerae* are “non-O1/O139” environmental strains that may or may not cause some form of gastroenteritis (Baker-Austin et al., 2018). *V. cholerae* strains that do cause cholera can be differentiated from other strains by the production of cholera toxin (CT) and toxin coregulated pilus (TCP) (Faruque et al., 1998). The disease is characterized by symptoms such as the classical profuse-watery diarrhea, known as “rice-water stool”, that leads to extreme dehydration, shock, and eventual death (Legros, 2018). This watery diarrhea is flecked with mucus, intestinal epithelial cells, and bacteria. A normal functioning mucus gel layer covering intestinal epithelial cells serves many functions, including as a dynamic defensive barrier against microbes both resident and foreign (Gaskins, 1997). The secretion of this mucus gel layer is the primary function of a specialized group of cells lining mucosal tissue, called goblet cells (Birchenough et al., 2015). Alteration of mucin production is hypothesized to occur in one of two ways: 1) by microbial factors that either modulate the secretion and synthesis of mucin, or by altering the chemical composition of this mucin, or 2) host factors that are released by local epithelial cells or immune cells in response to intestinal microbes (Belley et al., 1999; Mack et al., 1999). Large secretions of mucus are one result of *V. cholerae* infection and partially due to the effects of CT that lead to a release of massive amounts of mucin *via* a cAMP-dependent mechanism (Yardley et al., 1972; Lencer et al., 1990). *V. cholerae* has also been shown to penetrate mucus layers using its flagellum, while nonmotile Vibrios are significantly less efficient at colonization or even avirulent (Jones, 1977). Another study reported that *V. cholerae* outer membrane vesicles (OMVs) led to the priming of CD4^+^ T cells toward an inflammatory T helper 2 (Th2) response and led to expression of the cytokines IL-4, IL-13, and IL-17, all of which have been shown to lead to an upregulation in the production of mucin (Dabbagh et al., 1999; Shim et al., 2001; Chen et al., 2003; Chatterjee and Chaudhuri, 2013).

The effect of *V. cholerae* and cholera toxin on cellular immunity appears to be one that leads to CD4^+^ T cell differentiation to both Th1 and Th2 cell lineages, while Th2 cell types may be favored. Stimulation of T cells by intracellular bacteria generally leads to differentiation towards the Th1 cell lineage and a cellular-mediated response, which is characterized by activating phagocytes such as macrophages and cytotoxic T cells (CD8^+^ T cells), rather than antibody production. Th2 cells, on the other hand, are generally activated by extracellular pathogens and lead to a humoral immune response, characterized by B cell activation and immunoglobulin production (Zhu and Paul, 2008). In a report documenting T cell responses in Bangladeshi children, naturally infected cholera patients were shown to have elevated levels of both Th1 and Th2 cells, while those vaccinated had more modest increases in Th1 cells, indicating that both age as well as route of immunization likely play a factor in T cell populations (Arifuzzaman et al., 2012). Meanwhile, Xu-Amano et al. showed that oral immunization with CT preferentially led to the induction of Th2 cells, with some Th1-type cells still being detected (Xu-Amano et al., 1994).

As previously mentioned, one cytokine produced by Th2 cells is IL-13. A major effect of IL-13 is in inducing goblet cell differentiation, resulting in the production of excess mucus, a well characterized result of cholera disease (Wynn, 2003). One report by Bhuiyan *et al.* documented increases in this cytokine after stimulating lymphocytes isolated from hospitalized cholera patients. However, to date, work investigating Th2 cell production of IL-13 during *V. cholerae* infection is lacking (Bhuiyan et al., 2009). Another effect of Th2 cell responses is ultimately in B cell activation and immunoglobulin production. In humans, *V. cholerae* infection has been shown to lead to increases in IgM, IgG, and IgA (Levine et al., 1981; Yang et al., 2019). In the present study, we utilized a well-established *V. cholerae* zebrafish model. Though the zebrafish and human immune systems are largely the same, differences in immunoglobulins are present, as fish lack homologues of IgG and IgE (Gomez et al., 2013). Despite this, the mucosal tissue associated antibodies IgM and IgA (termed IgZ in zebrafish) are still present in fish, and likely have roles in teleost defense against *V. cholerae* infection.

While much research has gone into understanding cholera immunology and *V. cholerae* pathogenesis, many questions remain in the field. Perhaps the most important question remains how to create more efficacious cholera vaccines, as current vaccine efficacies are only ~60% (Shaikh et al., 2020). Because Th1 and Th2 responses are antagonistic, with cytokines from one preventing the development of the other, fully understanding the *V. cholerae* factors that elicit a Th1 vs. Th2 mediated response are therefore essential in improving vaccine efficacy (Rosenthal and Zimmerman, 2006). Our current understanding of CD4^+^ T cell responses is lacking, as is knowledge of the downstream cytokines and effector functions produced by these cells during cholera.

Furthermore, the zebrafish model is still in its infancy in characterizing and understanding the immune response to *V. cholerae* infection. Defining cell-mediated and humoral antibody responses will add to the cholera immunology knowledge base as a whole and will open the door for the fish model to be used in answering other unresolved questions in immune responses to *V. cholerae* infection.

In this work, we define key parts of the zebrafish immune response to *V. cholerae*, including cell mediated and humoral immunity, as well as a potential cytokine source of mucus production. Our results provide evidence for use of the zebrafish model as a tool for further immunological studies with the ultimate goal of developing more efficacious cholera vaccines.

## Materials and Methods

### Bacterial Strains and Culture Conditions

*V. cholerae* environmental strain 25493 (Sm^r^ [100 µg/ml]) and *V. cholerae* El Tor strain E7946 (Sm^r^ [100 µg/ml]) were used in this study. Bacterial strains were frozen in 15% glycerol in Luria-Bertani (LB) broth (Difco, NJ, USA) at -80°C. For experimentation, each strain was then grown in LB broth (Difco, NJ, USA) at 37°C under shaking conditions (180 rpm) or on plates in LB agar (Difco, NJ, USA) with the appropriate antibiotic(s). Thiosulfate-citratebile-sucrose (TCBS) agar (Difco, NJ, USA) was used as selective media for *V. cholerae*.

### Zebrafish

Wild-type AB zebrafish were used for all experiments. Zebrafish were housed in an automated recirculating tank system (Aquaneering, CA, USA) using water filtered by reverse osmosis and maintained at pH 7.0 to 7.5. The tank water was conditioned with Instant Ocean salt (Aquarium Systems, OH, USA) to a conductivity of 600 to 700 S. Zebrafish were euthanized in 100 ml of 32-µg/ml Tricaine-S (tricaine methane sulfonate; MS-222 [Western Chemical, WA, USA]) for a minimum of 25-30 min after cessation of opercular movement. All animal protocols were approved by the Wayne State University IACUC.

### Adult Zebrafish Infection Procedure

For experimental groups, 4-5 zebrafish were placed into a 400 ml beaker with perforated lids, containing 200 ml of tank water (autoclaved ddH2O with 60 mg/liter of Instant Ocean aquarium salts). Bacterial cultures were grown in LB broth at 37°C for 16 to 18 h with aeration. Bacteria were then washed once in phosphate-buffered saline (PBS) and diluted to a concentration of 10^9^ CFU/ml by measuring the OD at 600 nm. PBS diluted bacteria were then added directly to beakers to an infection concentration of 2.5 x 10^7^ CFU/ml and plated using serial dilutions for verification. Control fish were exposed to 1 ml of 1X PBS. Beakers containing fish were then placed in a glass-front incubator at 28°C for the duration of the experiment.

### Intestinal Colonization Assessment

At specified time points, fish were euthanized using tricaine as described above. Intestines were aseptically removed and placed in homogenization tubes (2.0 ml screw-cap tubes; Sarstedt, Nümbrecht, Germany) with 1.5 g of 1.0 mm glass beads (BioSpec Products, Inc., Bartlesville, OK) and 1 ml of 1x PBS, and held on ice. Homogenization tubes were loaded into a Mini-Beadbeater-24 (BioSpec Products, Inc.). Serial dilutions of homogenized tissue were performed using 1X PBS and the dilutions were plated onto LB agar plates with appropriate antibiotics.

### RNA Isolation and qRT-PCR

Intestinal tissue was homogenized in 1 ml 1x PBS using homogenization beads as described above. RNA was then extracted using Qiagen RNeasy Mini Kit (Qiagen, Hilden, Germany). Total RNA was resuspended in RNase-free water and quantified using a NanoDrop. cDNA was then synthesized using Invitrogen SuperScript III First-Strand Synthesis System cDNA kit (Invitrogen, Waltham, MA) from a specific amount of RNA. qRT-PCR was performed using SYBR green on a 7500 Real-Time PCR System (Applied Biosystems, Foster City, CA). Quantification of gene expression was determined using the comparative ^ΔΔ^CT method. Gene expression was normalized to the endogenous reference β-actin level and was reported as fold change relative to the reference gene. qPCR primer sequences for control and genes of interest are as follows: β-actin (F: 5’ TGCTGTTTTCCCCTCCATTG 3’) (R: 5’ TTCTGTCCCATGCCAACCA 3’), T-bet (F: 5’ AAATCCAGGAGCATGGACAG 3’) (R: 5’ TGAGACTGGATGTGGGTTTG 3’), GATA3 (F: 5’ CTGATAGGTGGGTCCTCTTC 3’) (R: 5’ CCGTTCATCTTGTGGTAAAG 3’), IL-13 (F: 5’ GTAGAGGAGGAGTCGGACTG 3’) (R: 5’ TCTAGTCCTCAGTGCGACGA 3’), IgM (F: 5’ GAAGCCTCCAATTCTGTTGG 3’) (R: 5’ CCGGGCTAAACACATGAAG 3’), IgZ (F: 5’ GAACCAAACTCAGGGTTGGA 3’) (R: 5’ CACCCAGCATTCTACAGCAA 3’).

### Mucin Determination *via* Microtiter PAS Assay

Mucin concentrations from excreted water were determined as previously described (Pukatzki et al., 2006; Nag et al., 2018). 100 µl/well of blank or mucin standards were loaded in a 96-well plate (Corning Costar; Corning, NY, USA), along with triplicates of samples. 0.1% periodic acid solution was added at 50 µl/well and mixed, then covered and incubated for 1 hr in a 37°C incubator. The plate was allowed to cool to room temperature, then 100 µl/well of Schiff’s reagent (Sigma-Aldrich) was added, mixed, and shaken for 15 minutes. Absorbance was then read at O.D. = 560 nm (Tecan Spectra Fluor plus; Tecan, Männedorf, Switzerland). The effective OD of test samples was calculated by subtraction of the PBS controlled (uninfected) fish excreted water OD from the test (infected) fish excreted water OD.

### Statistical Analysis

Experiments were performed in triplicate on separate occasions, unless otherwise specified. Data shown are presented as the mean ± standard deviation (SD). All statistical analyses, t-tests and two-way ANOVAs were performed using Prism version 7.0 for Windows (GraphPad Software, La Jolla, CA).

## Results

### Cell-Mediated Immune Response

**Figure 1:**
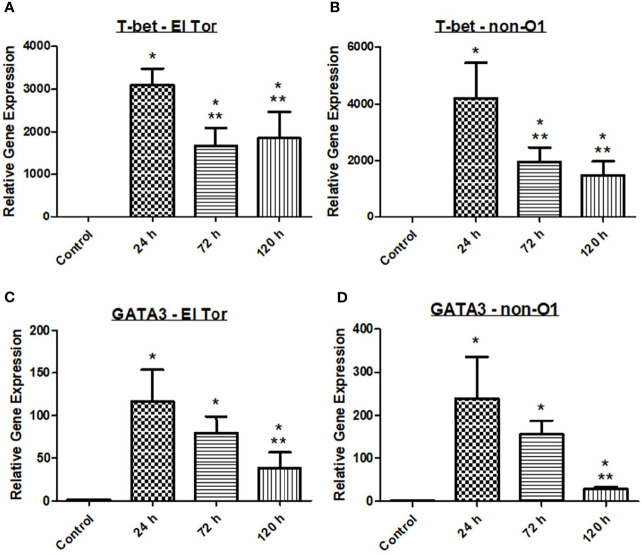
Adaptive immune responses in zebrafish against *V. cholerae* infection. WT zebrafish (n=5) were infected with E7946 (El Tor) or 25493 (non-O1) strains of *V. cholerae* at 2.5 x 10^7^ CFU/mL and then sacrificed at the indicated time points. **(A)** T-bet expression in fish infected with El Tor strain E7946. **(B)** T-bet expression in fish infected withnon-O1 strain 25493. **(C)** GATA3 expression in fish infected with El Tor strain E7946. **(D)** GATA3 expression in fish infected with non-O1 strain 25493. T-bet and GATA3 gene expression levels in zebrafish mRNA were determined through qRT-PCR. Gene expression was normalized against β-actin and expressed as fold change. Error bars indicate standard deviation. Data shown is from three experiments. *P < 0.05 as compared to control, **P < 0.05 as compared to 24 h infection.

With *V. cholerae* infection occurring extracellularly along the villi of the intestinal tract, we hypothesized that a Th2 mediated response would be seen in the zebrafish model, followed by a humoral-mediated response resulting in antibody production. To assess the zebrafish immune response to *V. cholerae*, we inoculated five fish *via* immersion using 2.5x10^7^ CFU/ml of either pandemic O1 strain E7946 (labelled “El Tor”) or environmental strain 25493 (labelled “non-O1”). Fish were then incubated and sacrificed at 24 h post infection (hpi), 72 hpi, or 120 hpi. After euthanasia, RNA was isolated from fish intestines and qPCR was used to assess the gene expression of transcription factors T-bet and GATA3 for each of these cell types. At all three time points after infection both T-bet and GATA-3 gene expression were significantly increased in zebrafish infected with either of the *V. cholerae* strains as compared to uninfected control fish (**Figure 1**). These data would support previous reports indicating that both Th1 and Th2 types of CD4^+^ T helper cells respond during *V. cholerae* infection.

### IL-13 Associated Mucin Production

**Figure 2:**
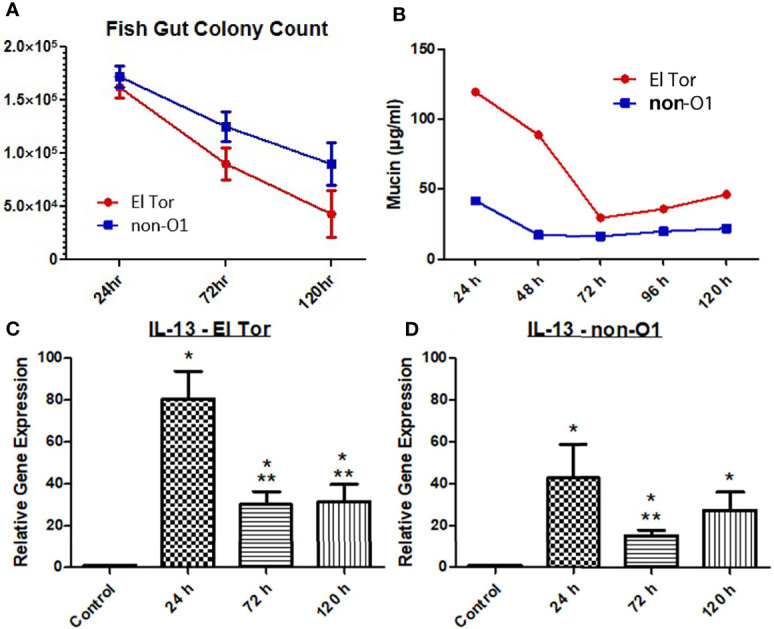
Mucin and IL-13 levels increase during *V. cholerae* infection. WT zebrafish (n=5) were infected with E7946 (El Tor) or 25493 (non-O1) strains of *V. cholerae* at 2.5 x 10^7^ CFU/mL and then sacrificed at the indicated time points, or water was taken during indicated time points. **(A)** CFU of the indicated *V. cholerae* strain taken from zebrafish intestines. **(B)** Mucin levels in the water of zebrafish infected with the indicated *V. cholerae* strain. **(C)** IL_13 expression in fish infected with El Tor strain E7946. **(D)** IL-13 levels in fish infected with non-O1 strain 25493. Mucin levels were determined *via* microtiter PAS assay. mRNA levels were determined through qRT-PCR. Gene expression was normalized against β-actin and expressed as fold change. Error bars indicate standard deviation. Data shown is from three experiments. *P < 0.05 as compared to control, **P < 0.05 as compared to 24 h infection.

We next wanted to explore if Th2 associated cytokine responses correlated with increased levels of mucin production. To investigate this, we first measured the intestinal colonization and mucin output from infected zebrafish tank water using the previously described microtiter PAS assay. We then chose to assess the Th2 associated cytokine IL-13, which has been shown to be elevated in response to *V. cholerae* infection and is well documented to induce goblet cell differentiation and mucin production (Bhuiyan et al., 2009; Chatterjee and Chaudhuri, 2013). Five fish were inoculated with 2.5×10^7^ cfu/ml of either El Tor or non-O1 *V. cholerae via* bath inoculation, followed by enumeration of *V. cholerae* in the intestinal tract 24 h, 72 h and 120 h post infection. For both strains the colonization levels were ~2×10^5^, 1×10^5^ and 5×10^4^ after 24, 72 and 120 hpi respectively (**Figure 2A**). The non-O1 strain showed slightly higher gut colonization than the El tor strain. Mucin output was elevated in fish infected with both strains, with the highest level of mucin output observed at 24 h. However, mucin levels were roughly 2.5 times higher in the El Tor strain as compared to the environmental non-O1 strain (**Figure 2B**).

Levels of mRNA expression for the cytokine IL-13 were significantly increased for all three time points by both *V. cholerae* strains, with 24 h being the time point of highest expression, then decreasing and plateauing at 72 h and 120 h, following the same pattern as mucin measurements (**Figures 2C, D**). Relative IL-13 gene expression was roughly 2 times higher in fish infected with the El Tor strain as compared to the environmental strain, which paralleled mucin measurements. Though IL-13 is not the only documented molecule that may influence mucin production, these results indicate that IL-13 patterns appear to correlate with mucin measurements in the same strain and when compared across strains to higher levels of IL-13 mRNA and mucin output both in the El Tor strain.

### Humoral Immune Response

**Figure 3:**
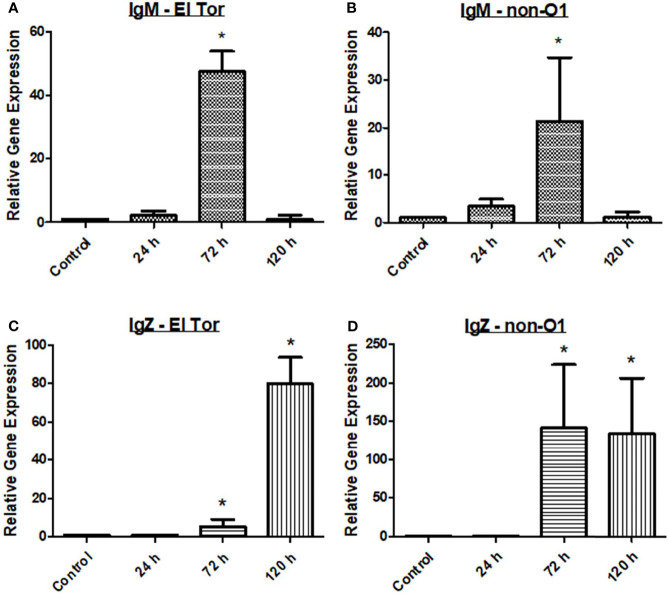
IgM and IgZ levels increase during *V. cholerae* infection. WT zebrafish (n=5) were infected with E7946 (El Tor) or 25493 (non-O1) strains of *V. cholerae* at 2.5 x 10^7^ CFU/mL and then sacrificed at the indicated time points. **(A)** IgM expression in fish infected with El Tor strain E7946. **(B)** IgM expression in fish infected with non-O1 strain 25493. **(C)** IgZ expression in fish infected with El Tor strain E7946. **(D)** IgZ expression in fish infected with non-O1 strain 25493. mRNA levels were determined through qRT-PCR. Gene expression was normalized against β-actin and expressed as fold change. Error bars indicate standard deviation. Data shown is from three experiments. *P < 0.05 as compared to control.

Finally, we wanted to assess the humoral response of the zebrafish to *V. cholerae* infection by measuring relevant antibody responses. Three immunoglobulins are present in the zebrafish, which are homologues of human IgM, IgD, and IgA, the latter known as IgZ/T in fish. Currently, the role of IgD is not well understood in teleosts, and current knowledge of human IgD indicates it likely does not play a large role in *V. cholerae* immunity. Therefore, mRNA levels of IgM and IgZ were measured in the zebrafish model during *V. cholerae* infection. Because IgM is well known to be an early responding immunoglobulin, we hypothesized that our model would show increases in this antibody first, with mRNA levels increasing slightly at 24 h, and reaching significantly increased levels by 72 h (**Figures 3A, B**). By 120 h, mRNA of IgM had fallen to baseline levels, and these patterns held true for fish infected with either *V. cholerae* strain. IgZ, the mucosal homologue of mammalian IgA, was significantly increased at 72 hpi and 120 hpi. However, 72 hpi IgZ levels were much lower in fish infected with the El Tor strain than in fish infected with the environmental non-O1 strain (**Figures 3C, D**). These data support previously documented immunoglobulin responses in humans, indicating that IgM and mucosal antibodies are important adaptive responses during *V. cholerae* infection.

## Discussion

Previous reports have indicated that there may be roles for both Th1 and Th2 cells in response to *V. cholerae* infection, while other reports have favored a stronger Th2 response (Xu-Amano et al., 1994; Zhu and Paul, 2008; Arifuzzaman et al., 2012). It has been well documented that Th2 responses generally occur during extracellular pathogenic infections. Additionally, Th2 cells are known activators of humoral immunity resulting in immunoglobulin production, and mucosal antibodies as well as IgG have been shown to be present in cholera patients (Xu-Amano et al., 1994; Zhu and Paul, 2008; Arifuzzaman et al., 2012). T-bet and GATA3 act through distal elements to control expression of immune regulator genes. The transcription factor T-bet is essential for Th1 cell mediated responses whereas GATA3 is responsible for both Th1 and Th2 cell mediated immune responses. In the *V. cholerae* zebrafish model, mRNA gene expression of both T-bet and GATA3 was significant increased at all three time points after *V. cholerae* infection, suggesting that both pandemic and non-pandemic *V. cholerae* strains (El Tor and non-O1) can induce Th1 and Th2 cell mediated adaptive immune responses following infection (**Figure 1**).

While some evidence in the literature does exist indicating Th2 cell lineages are favored in response to cholera, there is far from a consensus on cell mediated immunity. Many reports of CT being an immunomodulatory agent that favors Th2 cell differentiation are available. A study from Lavelle et al. shows that in mice, CT modulates dendritic cell (DC) cytokine production to promote Th2 and regulatory type 1 T cells (Tr1), which ultimately inhibit Th1 cell differentiation (Lavelle et al., 2004). Meanwhile, Mattsson *et al.* reported that DCs primed CD4^+^ T cells independent of Th1 associated cytokines, and resulted in a substantial generation of Th1, Th2, and Th17 cells (Mattsson et al., 2015). Other reports such as those by Chatterjee *et al.* indicate that other *V. cholerae* associated molecules, such as OMVs, favor a Th2 mediated response (Chatterjee and Chaudhuri, 2013). While the strain of *V. cholerae* causing infection and subsequently the virulence factors utilized during the infection may influence the CD4^+^ differentiation, data presented here do not indicate differences in responses, as one major difference between the strains used is the presence of CT. Zebrafish homologues to human dendritic cells have only recently been identified and are amongst the least investigated and understood immunological cell type in zebrafish to date (Lugo-Villarino et al., 2010). Future work documenting DC and Th17 cell responses in zebrafish may further the understanding of these cell types and provide clarity as to cholera cell mediated responses.

One hallmark symptom of cholera is profuse-watery diarrhea. This rice-water stool is mucus laden - partially a function of CT, which leads to increases in intracellular cAMP, leading to intestinal goblet cells activating cAMP response element binding protein (CREB) and ultimately massive amounts of mucin secretion (Yardley et al., 1972; Lencer et al., 1990). Previously, the zebrafish model has been shown to produce diarrhea and replicate the infectious cycle of cholera. Mitchell *at al.* reported increases in mucin filled goblet cells, as well as increases in mucin and diarrhea in fish infected with either El Tor or environmental non-O1 strains of *V. cholerae* (Mitchell et al., 2017). High colonization levels by two *V. cholerae* strains was observed up to 120 hpi in the current study. We were able to relate the production of mucin in the zebrafish gut with the degree of colonization using both an El Tor and an environmental non-O1 strain (**Figures 2A, B**). In addition, mRNA levels of the cytokine IL-13 were significantly increased at all three time points (**Figures 2C, D**).

Though mucin production is well known to occur during *V. cholerae* infection, and IL-13 production is established as an inducer of goblet cell differentiation and mucus secretion, evidence linking these together during cholera is currently lacking. While IL-13 levels have been assessed, these studies are generally done in investigations of CD4^+^ cell responses to cholera. In this study, data document an increase in both mucin production and the IL-13 gene expression level in the zebrafish, allowing for further investigation into this relationship using this model. One way the zebrafish model is uniquely suited to exploring these questions is by using genetic knockdowns in zebrafish. Morpholinos (MO) have been widely used to mediate gene knockdowns in many zebrafish models. Techniques such as these will enable experiments that determine IL-13 and other Th2 associated cytokines such as IL-4 or IL-17 involvement in mucus production.

In humans, the immunoglobulins IgM, IgG, and IgA have been shown to be elevated either during initial *V. cholerae* infection, or during secondary challenge (Levine et al., 1981; Yang et al., 2019). While zebrafish lack IgG, they possess homologues of the important mucosal antibodies IgM and IgA that play a vital role in defense of gastrointestinal pathogens like *V. cholerae* (Gomez et al., 2013). IgM is known as the first responding antibody, while it also has the ability to bind to the polyimmunglobulin receptor (pIgR), leading to its secretion into mucosal surfaces such as the lumen of the gut. In zebrafish infected with *V. cholerae*, IgM mRNA levels were slightly elevated at 24 hpi, significantly elevated at 72 hpi, and returning to baseline by 120 hpi (**Figures 3A, B**). For IgZ, the zebrafish mucosal immunoglobulin homologue of human IgA, mRNA gene expression was significantly increased at both 72 hpi and 120 hpi for fish infected with either *V. cholerae* strain (**Figures 3C, D**). Because Th2 responses ultimately lead to a humoral mediated immune response, elevations in mucosal associated immunoglobulins were expected based on significantly increased Th2 responses in the zebrafish (**Figures 1C, D**).

The data presented here further solidify the zebrafish model as a useful tool in the study of *V. cholerae* infection. By documenting the basic immune response of fish to *V. cholerae*, larger, more pressing questions can be answered. Currently, the literature tells a conflicting story of the nature of cell mediated immune responses during cholera. This work adds to that story by indicating that both Th1 and Th2 cells likely have a role in cholera defense. Future work documenting the zebrafish dendritic cells, as well as Th17 cells, may provide further clarity to the convoluted nature of this problem. In addition, this study provides a direct link between excess mucus production during cholera and IL-13 cytokine responses. By establishing this link, future experiments involving genetically modified zebrafish may prove useful in unearthing the role IL-13 and other Th2 associated cytokines play in excess mucus secretion. Finally, immunoglobulin responses in this work may aid understanding long term immunity and mucosal defenses that occur during cholera.

In sum, this study lays the groundwork for the use of the zebrafish as a relevant model for studying immunological questions in the cholera field. Use of this model will aid in answering looming immunology questions in pursuit of developing more efficacious cholera vaccines.

## Data Availability Statement

The original contributions presented in the study are included in the article/supplementary material. Further inquiries can be directed to the corresponding author.

## Ethics Statement

The animal study was reviewed and approved by Wayne State University IACUC.

## Author Contributions

DF designed and performed experiments, analyzed data, and wrote the manuscript. DN and JW aided in experimental design, data analysis and edited the manuscript. All authors contributed to the article and approved the submitted version.

## Funding

This work was supported by PHS grant R01AI127390 (to JW).

## Conflict of Interest

The authors declare that the research was conducted in the absence of any commercial or financial relationships that could be construed as a potential conflict of interest.

## Publisher’s Note

All claims expressed in this article are solely those of the authors and do not necessarily represent those of their affiliated organizations, or those of the publisher, the editors and the reviewers. Any product that may be evaluated in this article, or claim that may be made by its manufacturer, is not guaranteed or endorsed by the publisher.

## References

[B1] Ali M. Nelson A. R. Lopez A. L. Sack D. A. (2015). Updated Global Burden of Cholera in Endemic Countries. PloS Neglect. Trop. Dis. 9 (6), e0003832. doi: 10.1371/journal.pntd.0003832 PMC4455997 26043000

[B2] Arifuzzaman M. Rashu R. Leung D. T. Hosen M. I. Bhuiyan T. R. Bhuiyan M. S.. (2012). Antigen-Specific Memory T Cell Responses After Vaccination With an Oral Killed Cholera Vaccine in Bangladeshi Children and Comparison to Responses in Patients With Naturally Acquired Cholera. Clin. Vaccine Immunol. 19 (8), 1304–1311. doi: 10.1128/CVI.00196-12 22739692 PMC3416086

[B3] Baker-Austin C. Oliver J. D. Alam M. Ali A. Waldor M. K. Qadri F.. (2018). Vibrio Spp. Infections. Nat. Rev. Dis. Primers 4 (1), 1–19. doi: 10.1038/s41572-018-0005-8 30002421

[B4] Baker-Austin C. Trinanes J. A. Taylor N. G. Hartnell R. Siitonen A. Martinez-Urtaza J. (2013). Emerging Vibrio Risk at High Latitudes in Response to Ocean Warming. Nat. Climate Change 3 (1), 73–77. doi: 10.1038/nclimate1628

[B5] Belley A. Keller K. Göttke M. Chadee K. Göettke M. (1999). Intestinal Mucins in Colonization and Host Defense Against Pathogens. Am. J. Trop. Med. Hyg. 60 (4_suppl), 10–15. doi: 10.4269/ajtmh.1999.60.10 10344672

[B6] Bhuiyan T. R. Lundin S. B. Khan A. I. Lundgren A. Harris J. B. Calderwood S. B.. (2009). Cholera Caused by Vibrio Cholerae O1 Induces T-Cell Responses in the Circulation. Infect. Immun. 77 (5), 1888–1893. doi: 10.1128/IAI.01101-08 19237532 PMC2681774

[B7] Birchenough G. M. Johansson M. E. Gustafsson J. K. Bergström J. H. Hansson G. C. (2015). New Developments in Goblet Cell Mucus Secretion and Function. Mucosal Immunol. 8 (4), 712–719. doi: 10.1038/mi.2015.32 25872481 PMC4631840

[B8] Chatterjee D. Chaudhuri K. (2013). Vibrio Cholerae O395 Outer Membrane Vesicles Modulate Intestinal Epithelial Cells in a NOD1 Protein-Dependent Manner and Induce Dendritic Cell-Mediated Th2/Th17 Cell Responses. J. Biol. Chem. 288 (6), 4299–4309. doi: 10.1074/jbc.M112.408302 23275338 PMC3567681

[B9] Chen Y. Thai P. Zhao Y. H. Ho Y. S. DeSouza M. M. Wu R. (2003). Stimulation of Airway Mucin Gene Expression by Interleukin (IL)-17 Through IL-6 Paracrine/Autocrine Loop. J. Biol. Chem. 278 (19), 17036–17043. doi: 10.1074/jbc.M210429200 12624114

[B10] Dabbagh K. Takeyama K. Lee H. M. Ueki I. F. Lausier J. A. Nadel J. A. (1999). IL-4 Induces Mucin Gene Expression and Goblet Cell Metaplasia *In Vitro* and *In Vivo* . J. Immunol. 162 (10), 6233–6237. 10229869

[B11] Deeb R. Tufford D. Scott G. I. Moore J. G. Dow K. (2018). Impact of Climate Change on Vibrio Vulnificus Abundance and Exposure Risk. Estuaries. Coasts. 41 (8), 2289–2303. doi: 10.1007/s12237-018-0424-5 31263385 PMC6602088

[B12] Faruque S. M. Albert M. J. Mekalanos J. J. (1998). Epidemiology, Genetics, and Ecology of Toxigenic Vibrio Cholerae. Microbiol. Mol. Biol. Rev. 62 (4), 1301–1314. doi: 10.1128/MMBR.62.4.1301-1314.1998 9841673 PMC98947

[B13] Gaskins H. R. (1997). Immunological Aspects of Host/Microbiota Interactions at the Intestinal Epithelium. Gastrointest. Microbiol. 2, 537–587. doi: 10.1007/978-1-4757-0322-1_14

[B14] Gomez D. Sunyer J. O. Salinas I. (2013). The Mucosal Immune System of Fish: The Evolution of Tolerating Commensals While Fighting Pathogens. Fish. Shellfish. Immunol. 35 (6), 1729–1739. doi: 10.1016/j.fsi.2013.09.032 24099804 PMC3963484

[B15] Jones G. W. (1977). “The Attachment of Bacteria to the Surfaces of Animal Cells,” in Microbial Interactions (Boston, MA: Springer), 139–176.

[B16] Lavelle E. C. Jarnicki A. McNeela E. Armstrong M. E. Higgins S. C. Leavy O.. (2004). Effects of Cholera Toxin on Innate and Adaptive Immunity and its Application as an Immunomodulatory Agent. J. Leukocyte. Biol. 75 (5), 756–763. doi: 10.1189/jlb.1103534 14704372

[B17] Legros D. (2018). Global Cholera Epidemiology: Opportunities to Reduce the Burden of Cholera by 2030. J. Infect. Dis. 218 (suppl_3), S137–S140. doi: 10.1093/infdis/jiy486 30184102 PMC6207143

[B18] Lencer W. I. Reinhart F. D. Neutra M. R. (1990). Interaction of Cholera Toxin With Cloned Human Goblet Cells in Monolayer Culture. Am. J. Physiology-Gastrointest. Liver. Physiol. 258 (1), G96–G102. doi: 10.1152/ajpgi.1990.258.1.G96 2154122

[B19] Levine M. M. Black R. E. Clements M. L. Cisneros L. Nalin D. R. Young C. R. (1981). Duration of Infection-Derived Immunity to Cholera. J. Infect. Dis. 143 (6), 818–820. doi: 10.1093/infdis/143.6.818 7252264

[B20] Lugo-Villarino G. Balla K. M. Stachura D. L. Bañuelos K. Werneck M. B. Traver D. (2010). Identification of Dendritic Antigen-Presenting Cells in the Zebrafish. Proc. Natl. Acad. Sci. 107 (36), 15850–15855. doi: 10.1073/pnas.1000494107 20733076 PMC2936643

[B21] Mack D. R. Michail S. Wei S. McDougall L. Hollingsworth M. A. (1999). Probiotics Inhibit Enteropathogenic E. Coli Adherence *In Vitro* by Inducing Intestinal Mucin Gene Expression. Am. J. Physiology-Gastrointest. Liver Physiol. 276 (4), G941–G950. doi: 10.1152/ajpgi.1999.276.4.G941 10198338

[B22] Mattsson J. Schön K. Ekman L. Fahlen-Yrlid L. Yrlid U. Lycke N. Y. (2015). Cholera Toxin Adjuvant Promotes a Balanced Th1/Th2/Th17 Response Independently of IL-12 and IL-17 by Acting on Gsα in CD11b+ DCs. Mucosal Immunol. 8 (4), 815–827. doi: 10.1038/mi.2014.111 25425266

[B23] Mitchell K. C. Breen P. Britton S. Neely M. N. Withey J. H. (2017). Quantifying Vibrio Cholerae Enterotoxicity in a Zebrafish Infection Model. Appl. Environ. Microbiol. 83 (16), e00783-17. doi: 10.1128/AEM.00783-17 28625997 PMC5541206

[B24] Nag D. Mitchell K. Breen P. Withey J. H. (2018). Quantifying Vibrio Cholerae Colonization and Diarrhea in the Adult Zebrafish Model. JoVE (J. Visualized. Exp.) 137), e57767. doi: 10.3791/57767 PMC6126457 30059022

[B25] Pukatzki S. Ma A. T. Sturtevant D. Krastins B. Sarracino D. Nelson W. C.. (2006). Identification of a Conserved Bacterial Protein Secretion System in Vibrio Cholerae Using the Dictyostelium Host Model System. Proc. Natl. Acad. Sci. 103 (5), 1528–1533. doi: 10.1073/pnas.0510322103 16432199 PMC1345711

[B26] Rosenthal K. S. Zimmerman D. H. (2006). Vaccines: All Things Considered. Clin. Vaccine Immunol. 13 (8), 821–829. doi: 10.1128/CVI.00152-06 16893980 PMC1539119

[B27] Shaikh H. Lynch J. Kim J. Excler J. L. (2020). Current and Future Cholera Vaccines. Vaccine 38, A118–A126. doi: 10.1016/j.vaccine.2019.12.011 31879125

[B28] Shim J. J. Dabbagh K. Ueki I. F. Dao-Pick T. Burgel P. R. Takeyama K.. (2001). IL-13 Induces Mucin Production by Stimulating Epidermal Growth Factor Receptors and by Activating Neutrophils. Am. J. Physiology-Lung. Cell. Mol. Physiol. 280 (1), L134–L140. doi: 10.1152/ajplung.2001.280.1.L134 11133503

[B29] Vezzulli L. Grande C. Reid P. C. Hélaouët P. Edwards M. Höfle M. G.. (2016). Climate Influence on Vibrio and Associated Human Diseases During the Past Half-Century in the Coastal North Atlantic. Proc. Natl. Acad. Sci. 113 (34), E5062–E5071. doi: 10.1073/pnas.1609157113 27503882 PMC5003230

[B30] Wynn T. A. (2003). IL-13 Effector Functions. Annu. Rev. Immunol. 21 (1), 425–456. doi: 10.1146/annurev.immunol.21.120601.141142 12615888

[B31] Xu-Amano J. Jackson R. J. Fujihashi K. Kiyono H. Staats H. F. McGhee J. R. (1994). Helper Th1 and Th2 Cell Responses Following Mucosal or Systemic Immunization With Cholera Toxin. Vaccine 12 (10), 903–911. doi: 10.1016/0264-410X(94)90033-7 7975832

[B32] Yang J. S. An S. J. Jang M. S. Song M. Han S. H. (2019). IgM Specific to Lipopolysaccharide of Vibrio Cholerae Is a Surrogate Antibody Isotype Responsible for Serum Vibriocidal Activity. PloS One 14 (3), e0213507. doi: 10.1371/journal.pone.0213507 30845262 PMC6405115

[B33] Yardley J. H. Bayless T. M. Luebbers E. H. Halsted C. H. Hendrix T. R. (1972). Goblet Cell Mucus in the Small Intestine. Findings After Net Fluid Production Due to Cholera Toxin and Hypertonic Solutions. Johns. Hopkins. Med. J. 131 (1), 1–10. 5042530

[B34] Zhu J. Paul W. E. (2008). CD4 T Cells: Fates, Functions, and Faults. Blood 112 (5), 1557–1569. doi: 10.1182/blood-2008-05-078154 18725574 PMC2518872

